# Evaluation of Critical Dynamic Stress and Accumulative Plastic Strain of an Unbound Granular Material Based on Cyclic Triaxial Tests

**DOI:** 10.3390/ma14195722

**Published:** 2021-09-30

**Authors:** Qishu Zhang, Wuming Leng, Bin Zhai, Fang Xu, Junli Dong, Qi Yang

**Affiliations:** 1 School of Civil Engineering, Central South University, Changsha 410075, China; 174801020@csu.edu.cn (Q.Z.); wmleng@csu.edu.cn (W.L.); email1995zb@csu.edu.cn (B.Z.); dongjunli@csu.edu.cn (J.D.); qiyang123@csu.edu.cn (Q.Y.); 2 National Engineering Laboratory for High Speed Railway Construction, Central South University, Changsha 410075, China

**Keywords:** unbound granular materials, critical dynamic stress, accumulative plastic strain, cyclic triaxial test, BP neural network

## Abstract

Critical dynamic stress (*σ*_cri_) and accumulative plastic strain (*ε*_p_) are primary indicators regarding the dynamic stability of unbound granular materials (UGMs). This study aims to seek an effective method to evaluate the dynamic stability of UGMs used in railway subgrades. First, the dynamic characteristics of an UGM used in railway subgrade bed construction were investigated by performing a series of large-scale cyclic triaxial tests, with the results showing that *ε*_p_ versus cycle number (*N*) curves can be categorized into stable, failure, and critical patterns. Grey relational analyses were then established, where the analyzed results demonstrated that the *ε*_p_–*N* curve pattern and final accumulative plastic strain (*ε*_s_) of the stable curves are strongly correlated with the moisture content (*w*), confining pressure (*σ*_3_), and dynamic deviator stress (*σ*_d_). The analyzed grey relational grades distributed in a narrow range of 0.72 to 0.81, indicating that *w*, *σ*_3_, and *σ*_d_ have similar degrees of importance on determining the *ε*_p_–*N* curve patterns and the values of *ε*_s_ of the UGM. Finally, a data processing method using a back-propagation (BP) neural network is introduced to analyze the test data, and an empirical approach is developed to evaluate the *σ*_cri_ (considering the effects of *σ*_3_ and *w*) and *ε*_s_ (considering the effects of *σ*_3_, *w*, and *σ*_d_) of the UGM. The analyzed results illustrated that the developed method can effectively reflect the linear/non-linear relationships of *σ*_cri_ and *ε*_s_ with respect to *σ*_3_ and/or *σ*_d_. The *σ*_cri_ approximately increases linearly with increasing *σ*_3_, and a simple empirical formula is proposed for the *σ*_cri_. In addition, *ε*_s_ and its variation rate increase non-linearly with increasing *σ*_d_ but decrease non-linearly as *σ*_3_ increases.

## 1. Introduction

The serviceability of railway tracks is strongly associated with the dynamic stability of unbound granular layers atop weaker layers of the subgrades, of which the critical dynamic stress (*σ*_cri_) and accumulative plastic/permanent stain (*ε*_p_) are primary indicators regarding the dynamic stability of unbound granular materials (UGMs) [1,2,3,4,5,6]. Subgrades of railways accumulate permanent settlement under dynamic traffic loading, and excessive accumulative surface settlement typically causes problems, such as vehicle turbulence in transition sections [7,8], uneven settlement [9,10,11], and lateral expansion of embankment shoulders [12,13,14]. One of the most effective ways to control the accumulative deformation of subgrades is maintaining the traffic-induced dynamic stress to be less than a critical value (i.e., the *σ*_cri_), under which the development rate of *ε*_p_ gradually decreases and can ultimately cease with an increase in the dynamic loading cycles [5,15,16]. Furthermore, the rapid development of high-speed transport and heavy-haul freight demands the subgrades beneath track structures must satisfy strict requirements on accumulative permanent settlements [17,18,19]. Therefore, evaluations of the critical dynamic stress and accumulative plastic strain of the UGM layers are important in the design of a railway.

The cyclic triaxial test is an efficient way to study the critical dynamic stress of subgrade materials [20,21,22,23]. Qualitative analyses of critical dynamic stress of different types of materials showed that *σ*_cri_ is linked to the saturation degree, stress history, and dynamic loading path of the materials [15,24]. Cheung et al. [25] designated the stress level under which 1% accumulative permanent strain was generated within 1000 loading cycles as the *σ*_cri_. Frost et al. [26] presented that the *σ*_cri_ of fine-grained subgrades is approximately half of the deviator stress corresponding to static destruction. Wang et al. [27] reported that the initial stress state could considerably affect the *σ*_cri_ of a silty clay. Recently, a few empirical models were developed to predict the *σ*_cri_; Zhang et al. [28] developed a regression model for predicting the *σ*_cri_ based on the fuzzy linear regression theory and structural element method. Xu et al. [6] presented an empirical method to predict the *σ*_cri_ by introducing the concept of critical stress ratio; Zhai et al. [16] predicted the *σ*_cri_ of UGMs as a function of confining pressure based on results of cyclic triaxial tests of specimens with optimal moisture content. Most existing studies on *σ*_cri_ were qualitative investigations. Several evaluation models of *σ*_cri_ have also been proposed on the basis of cyclic triaxial tests; however, the factors considered in the models regarding the *σ*_cri_ of UGMs are incomplete, and systematic studies of *σ*_cri_ considering the effects of both moisture content and confining pressure are desirable.

The cyclic triaxial test is also commonly used to study the accumulative plastic deformation of UGMs. Generally, the *ε*_p_ versus cycle number (*N*) curves can be identified from a cyclic triaxial test. Instances of commonly used relationships are the power-function [29], semi-logarithmic [30], and hyperbolic models [31], which simply reflect the variation relationship between the *ε*_p_ and dynamic loading cycles; therefore, modifications have been proposed with considerations of the effects of stress path, static strength, confining pressure, etc. Li and Selig [32] extended the power function model while including the physical state and static strength of the tested soil; Chai and Miura [33] then extended the method of Li and Selig [32] by incorporating the initial static deviator stress (*σ*_si_); Wu et al. [34] improved the original power-function model while considering the effects of cyclic stress ratio. Moghaddas-Nejad and Small [35] improved the semi-logarithmic model by incorporating confining pressure and dynamic deviator stress; Stewart [36] extended the model by including the stress path; Xu et al. [37] developed a probabilistic method to predict *ε*_p_ of UGMs based on the semi-logarithmic model. In addition, a few models in different formulations were also proposed to predict the *ε*_p_. Lekarp et al. [38] formulated *ε*_p_ using the length of the stress path and shear stress ratio. El-Badawy and Witczak [39] predicted *ε*_p_ as a function of stress state, plasticity index, resilient response, saturation degree, *N*, fine contents, and material strength. Xiao et al. [40] proposed a unified prediction model of *ε*_p_ using the imaging-based morphological indexes of aggregate and concept of shear stress ratio.

The existing methods generally predict the *ε*_p_ well within small loading cycles. However, they may overestimate the *ε*_p_ of UGMs under large loading cycles because most of them demonstrate a monotonic increasing relationship between *ε*_p_ and *N*, which is contrary to the fact that the rate of *ε*_p_ of stable specimens gradually declines and finally ceases when the material enters a shakedown state [4,16,41]. Moreover, comparing with predicting the development trend of *ε*_p_, it is more practical to evaluate the final accumulative deformation of the UGM layers in the design of a railway subgrade.

In this study, the dynamic stability of an UGM under long-term dynamic loading was investigated by a series of large-scale cyclic triaxial tests. The main influencing factors and their influencing extent regarding the dynamic stability of the UGM were identified by grey relational analyses of the test results. Then, a data processing method in combination with a back-propagation (BP) neural network is introduced, from which an approach is developed to evaluate the *σ*_cri_ (considering the effects of *σ*_3_ and *w*) and final *ε*_p_ (*ε*_s_, considering the effects of *σ*_3_, *w*, and *σ*_d_) of the UGM, where the analyzed results demonstrated that the developed method can effectively describe the linear/non-linear behavior of *σ*_cri_ and *ε*_s_ regarding the confining pressure (*σ*_3_) and/or dynamic deviator stress (*σ*_d_).

## 2. Test Materials and Methods

**Figure 1:**
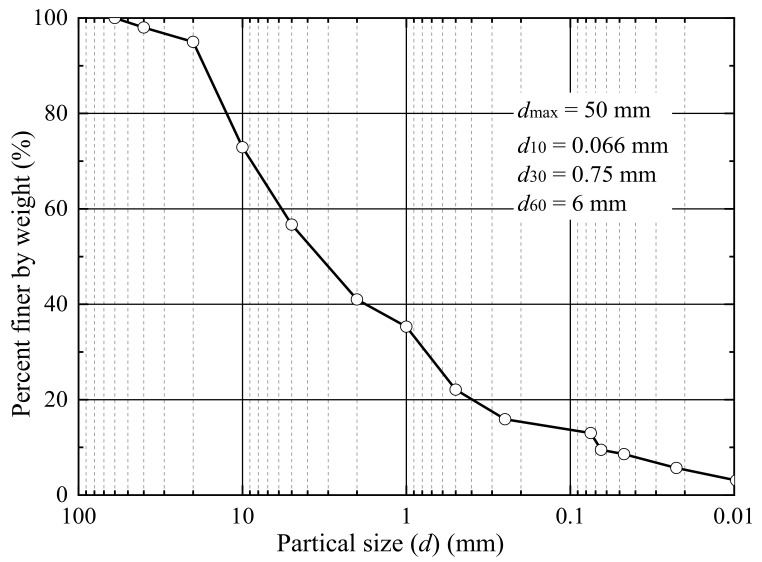
Particle size gradation curve of the UGM [16,37].

The tests were performed using a UGM consisted by fine particles, sand, and gravel. The particle size (*d*) gradation curve of the UGM is presented in Figure 1. The specific gradation properties of the UGM are *d*_60_ = 6 mm, *d*_30_ = 0.75 mm, *d*_10_ = 0.066 mm, *d*_max_ = 50 mm, *C*_c_ = 1.42, and *C*_u_ = 80, where *d*_60_, *d*_30_, *d*_10_, *d*_max_, *C*_c_, and *C*_u_ are the particle diameters corresponding to 60%, 30%, and 10% finer, maximum diameter, coefficient of curvature, and coefficient of uniformity, respectively. The tested UGM is categorized as a Group A subgrade bed filling in line with a railway standard of China [42] and belongs to the silty gravel group by following the Unified Soil Classification System of ASTM D2487-17e1 [43].

**Figure 2:**
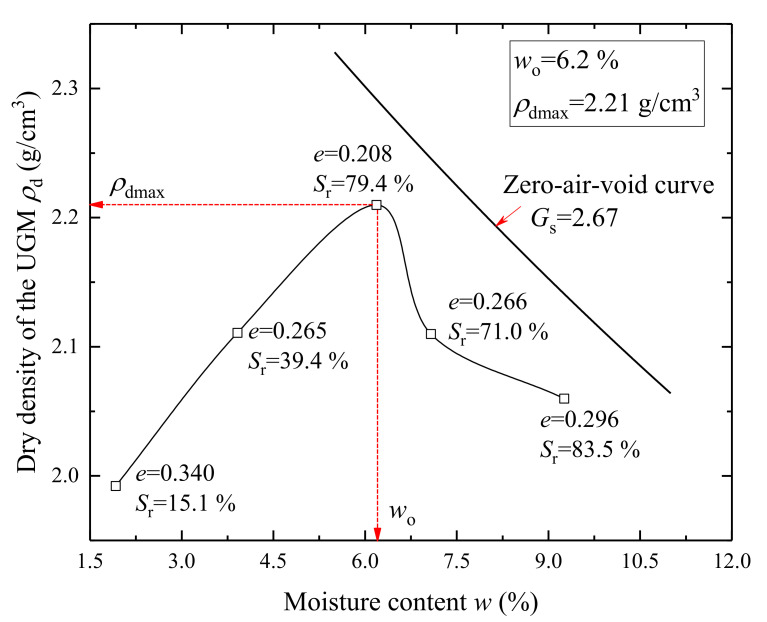
Compaction curve of the UGM.

The compaction curve of the UGM was tested by following the ASTM D1577-12 compaction standard using the modified proctor [44], as presented in Figure 2. In the compaction test, the UGM with different moisture contents was compacted using modified proctor with straightforward compaction energy, and the moisture contents after compaction were measured. Figure 2 also presents the zero air void curve of the UGM and basic physical parameters of each group of compacted material, including the specific gravity (*G*_s_), final void ratio (*e*), and final saturation degree (*S*_r_). Meanwhile, it can be determined from the compaction curve that the optimal moisture content (*w*_o_) and maximum dry density (*ρ*_dmax_) of the UGM are 6.2% and 2.21 g/cm^3^, respectively.

**Figure 3:**
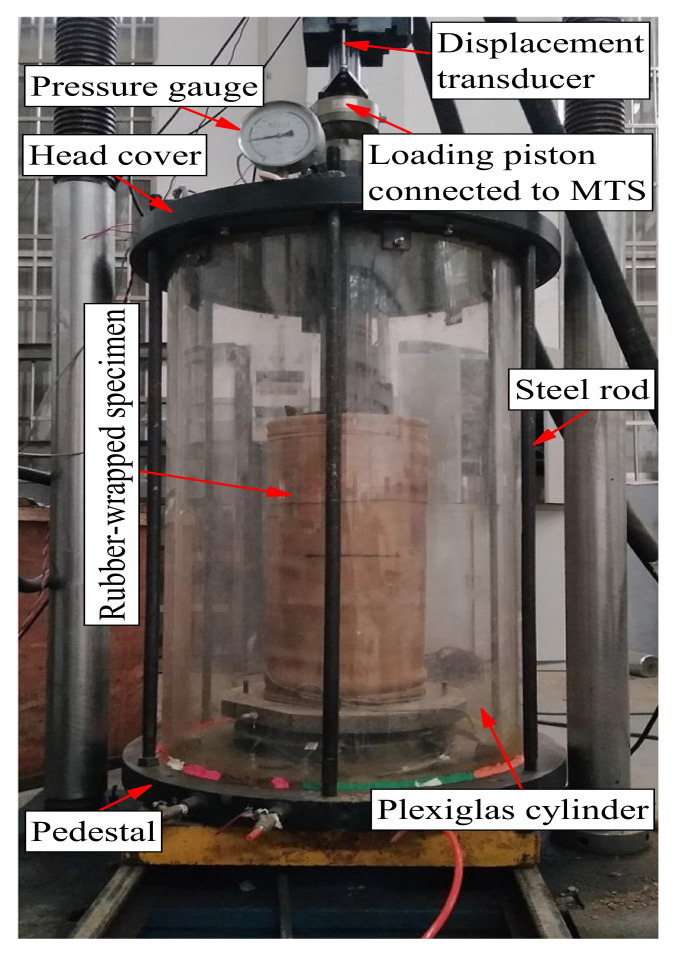
Photograph of the test device [16,37].

Large-scale cyclic triaxial test equipment was used to perform the tests, which mainly consisted of a confining cell and a loading frame. The loading frame can generate dynamic or monotonic loadings on the tested specimens, of which the maximum loading value is 50 kN; the test device can therefore conduct both cyclic and static triaxial tests. Figure 3 presents a photograph of the used test device, where the displacement gauge installed on the actuator has a precision of 0.01 mm, and variations in the specimen axil displacement during the test were recorded using a computer linked to a data logger.

The prominent advantage of the test equipment is that it can facilitate tests on subgrade soils with large particle sizes. According to the ASTM D7181-20 standard [45], the diameter of specimens is required to be no less than six times the *d*_max_ of the soil for reducing the size effects of specimens on the test data. Currently, a large proportion of triaxial devices are manufactured for conducting tests with soil specimen diameters less than 150 mm (e.g., 39.1, 50, 100 and 150 mm) [46,47,48,49]. However, the *d*_max_ of UGMs used as railway subgrade fillings is generally within the range of 50–60 mm as specified by a railway standard of China [42]. Therefore, regular cyclic triaxial devices cannot satisfy the requirement on the ratio of specimen diameter to *d*_max_. The used test equipment can facilitate tests of specimens with a diameter of 300 mm. The ratio of specimen diameter to *d*_max_ is therefore six in the present study, which satisfies the specification of ASTM D7181-20 standard [45].

Consistent compaction efforts were adopted in preparing the cyclic triaxial test specimens to ensure the reliability of test results. Similar to the compaction test, the UGM was first scattered, and then mixed by adding distilled water to reach the optimal moisture content and kept in a hermetic plastic bag to humidify for more than 48 h to homogenize the moisture content. The height and diameter of the specimens were 300 mm and 600 mm, respectively. To ensure the consistence of the prepared specimens, it was required to control the moist mass added, the thickness of each compacted layer, and the compaction efforts. In this study, the UGM with optimal moisture content was compacted into a steel specimen preparation mold by six layers, with each layer having the same moist mass of 16.065 kg. Then, each incompact layer was compacted to a target thickness of 100 mm using a 17 kg steel impact hammer with a drop distance of approximately 500 mm, to attain a target compaction degree of 0.97. It should be noted that keeping consistent layer moist mass added, consistent compaction layer thickness, and consistent impact hammer mass and drop distance (i.e., compaction efforts) is helpful to ensure the consistency of the triaxial UGM specimens.

Both optimal moisture content and saturation specimens were tested in the cyclic triaxial tests. The specimens with optimal moisture content were achieved by controlling the amount of distilled water added in the UGM, which corresponded to a saturation degree of 79.4%, as presented in Figure 2. The full saturation of UGM specimens was achieved by the following steps: (1) the specimens were firstly pre-saturated using vacuum suction for 2 h; (2) further saturation of the UGM specimens was achieved by injecting compressed carbon dioxide (CO_2_) and then distilled water from the bottom to the top of the specimens with a small confining pressure to reach a stationary flow state; (3) finally, back pressure was applied to reach a minimum pore pressure coefficient value of 0.95. Similar methods to ensure full saturation of specimens were also reported and recommended by Mehdizadeh [50].

**Figure 4:**
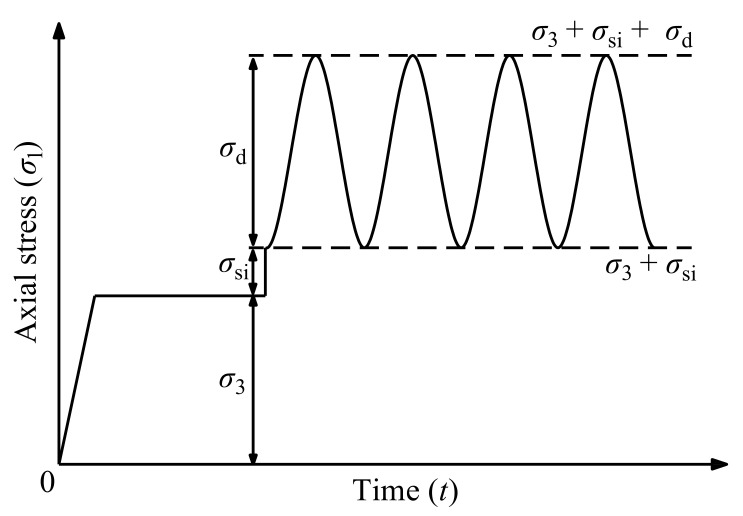
Cyclic loading curve [16,37].

In this study, 28 large-scale cyclic triaxial tests were performed, and the specific test schemes are listed in Table 1. Based on the field data reported by the China Academy of Railway Science, the maximum dynamic stress of railway subgrades is discrete in a range of 35–185 kPa when the train axle load is within 19.6–22.5 t [51]. Moreover, to consider a future developing trend that the heavy-haul train axle load will increase up to 35 t and investigate the *σ*_cri_ of the UGM, this study expanded the value range of the deviator stress *σ*_d_. Specifically, the range of *σ*_d_ for the tested saturation specimens and optimal moisture content specimens are 50–250 kPa and 100–400 kPa, respectively. Moreover, the operation speed of heavy-haul freight trains is much less than the normal and high-speed passenger trains; for instance, the operation speed of heavy-haul freight trains in China is mainly in the range of 20–80 km/h [52,53,54], and the freight train speed in Australia is generally within 40–80 km/h [55,56]. The wagon length of freight trains in China is 12–14 m [57]. In the cyclic triaxial tests, a 1 Hz sine-wave cyclic deviator stress was applied by closing the drainage valve, which corresponded to a typical operation speed of 50 km/h for the heavy-haul freight train in China. A 15 kPa initial static deviator stress (*σ*_si_) was applied prior to the application of *σ*_d_ [42], to simulate the vertical stress resulted by the rail, sleeper, and ballast. More detailed introduction regarding the test method and procedures can refer to the study of Xu et al. [37]. Figure 4 presents the schematic dynamic loading curve applied in the cyclic triaxial tests. The tests were terminated when the specimens were destroyed or the *ε*_s_ was achieved. Based on a few preliminary tests, the specimen was considered to be destroyed when the accumulative plastic strain reached 15%, and when the accumulative plastic strain was lower than 10% and its increment within 2 h was less than 1 mm, the specimen was deemed to attain a shakedown state in which the further plastic strain increments are negligible.

## 3. Results and Discussion of Accumulative Plastic Strain Curves

**Figure 5:**
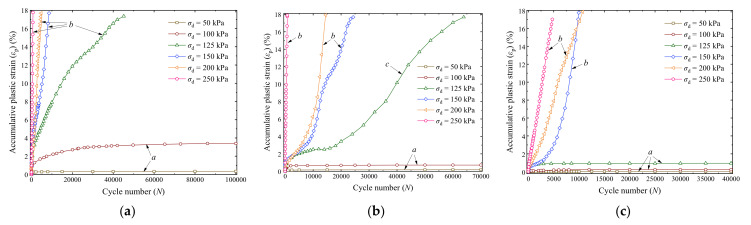
*ε*_p_—*N* relationship of saturation specimens: (**a**) *σ*_3_ = 15 kPa; (**b**) *σ*_3_ = 30 kPa; (**c**) *σ*_3_ = 60 kPa [16].

**Figure 6:**
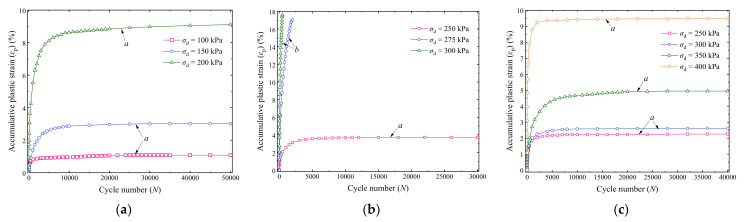
*ε*_p_—*N* relationship of specimens with optimal moisture content: (**a**) *σ*_3_ = 15 kPa; (**b**) *σ*_3_ = 30 kPa; (**c**) *σ*_3_ = 60 kPa.

Figure 5 and Figure 6 present the variation curves of the accumulative plastic strain *ε*_p_ with cycle number *N* of saturation and optimal moisture content specimens, respectively, where different curves correspond to different *σ*_d_.

It can be observed that the *ε*_p_ and its rate decline with an increase in *σ*_3_ but increase with the increase in *σ*_d_. Hence, the confining pressure and dynamic deviator stress have significant impacts on the development of *ε*_p_ of the UGM. The values of *ε*_p_ are generally greater than those reported in the existing literature [46,47,48,49]. One possible reason is that the tested confining pressures are relatively small comparing with existing studies. The low confining pressures tested in the present study can appropriately simulate the stress states of the subgrade soils in the field. The confining pressure of the subgrade soils can be approximately estimated as

*σ*_3_ = *K*_0_(*σ*_s*i*_ + *γh*) (1)

where *K*_0_ is the coefficient of lateral earth pressure and a value of 0.8 is generally adopted [58], *γ* is the unit weight of the subgrade soil and a value of 22 kN/m^3^ is generally acceptable, and *h* is the depth. The subgrade bed layers of a railway embankment generally have a thickness of 2.5 m [42]. As a result, the confining pressure of the subgrade bed layers is generally within the range of 12–56 kPa. Therefore, the confining pressures of 15, 30, and 60 kPa were selected to simulate the stress states of the UGM in the field.

Based on the variation tendencies of the accumulative plastic strains in Figure 5 and Figure 6, the *ε*_p_ versus *N* curves can be divided into three types, i.e., the stable (class a), failure (class b), and critical (class c) patterns. A stable pattern represents that the growth rate of *ε*_p_ gradually attenuates and finally ceases with the increase in loading cycles. Because of the internal erosion behavior, the fine particles can provide lateral support for the coarse particles, which leads to local particle rearrangement under dynamic loading and compression of the intergranular void [50,59], thus a densification effect would be formed. Finally, a stable UGM specimen enters a dense state in which it can resist the damage effects of the cyclic loading, and no further plastic strain is produced in subsequent loading cycles. By contrast, a failure pattern illustrates that the accumulative plastic strain of the specimen continuously rapidly increases to a *ε*_p_ value greater than 15% within small loading cycles or collapses under *σ*_d_. In addition, the *ε*_p_ versus *N* curve of a critical specimen is between the stable and failure patterns, of which the increase tendency of the accumulative plastic strain fluctuates and presents uncertainties with increasing the loading cycles. The dynamic deviator stress with respect to the critical curve pattern is generally designated as the critical dynamic stress *σ*_cri_ of an UGM.

## 4. Evaluation of Critical Dynamic Stress and Final Accumulative Plastic Strain

### 4.1. Evaluation of Critical Dynamic Stress

#### 4.1.1. Grey Relation of *ε*_p_–*N* Curve Pattern Regarding *w*, *σ*_3_, and *σ*_d_

It can be seen that the *ε*_p_ versus *N* curve pattern of the UGM is greatly affected by the dynamic deviator stress *σ*_d_, confining pressure *σ*_3_, and moisture content *w*, as presented in Figure 5 and Figure 6. In this section, the grey correlation theory was used to investigate the correlation degree of *ε*_p_–*N* curve patterns in regard to the *σ*_d_, *σ*_3_, and *w*.

Generally, principal component, regression, and variance analyses are commonly used to analyze systems. However, strict requirements should be met when using these methods, including (1) samples to obey typical probability distributions, (2) large size samples to ensure reliable analyses, and (3) a mass of calculations; moreover, the results between the qualitative and quantitative analyses are inconsistent at times, thereby occasionally leading to unreliable conclusions [60]. While the GRA is an advanced system analysis method and extensively employed in medical, economic, engineering, educational, and environmental fields to determine whether data sequences of a system have strong correlations. The method can effectively evaluate systems with small size data, where traditional statistical methods are unsuitable, because small size samples usually involve a great deal of grey information. Furthermore, the GRA has strong nonlinear fitting ability and convenient programming function [61]. The method can determine whether data sequences have strong correlations by comparing the similarity of their geometric curves, and greater similarities imply larger grey relational grades. The GRA does not have traditional demands on data distribution and can evaluate small size samples effectively, which would not generate disagreements between qualitative and quantitative results [60]. A brief introduction regarding the analysis steps of the GRA is as follows:

To perform GRA conveniently, non-dimensional treatment of the feature sequence (*Y* = (*y*_1_, *y*_2_, *y*_3_, …, *y*_n_)) and observation sequences (*X_i_* = (*x_i_*_1_, *x_i_*_2_, *x_i_*_3_, …, *x_i_*_n_), *i* = 1, 2, 3, …, *m*) is carried out by normalizing the sequences using the initial values as

(2) $${X^{\prime }}_{i}=X_{i}/x_{i1}=({x^{\prime }}_{i1},\;{x^{\prime }}_{i2},\;{x^{\prime }}_{i3},\ldots {x^{\prime }}_{in}),\;i=1,\;2,\;3,\ldots m$$

(3) $$Y^{\prime }=Y/y_{1}=({y^{\prime }}_{1},\;{y^{\prime }}_{2},\;{y^{\prime }}_{3},\ldots {y^{\prime }}_{n})$$

The grey correlation coefficient (*γ_ij_*, *i* = 1, 2, 3,…*m*, *j* = 1, 2, 3, …, *n*) between *y_j_* and *x_ij_* is calculated as

(4) $$\gamma_{ij}=\frac{\left[\underset{i=1}{\overset{m}{\min}}\underset{j=1}{\overset{n}{\min}}\left|{x^{\prime }}_{ij}-{y^{\prime }}_{j}\right|+\xi \underset{i=1}{\overset{m}{\max}}\underset{j=1}{\overset{n}{\max}}\left|{x^{\prime }}_{ij}-{y^{\prime }}_{j}\right|\right]}{\left[\left|{x^{\prime }}_{ij}-{y^{\prime }}_{j}\right|+\xi \underset{i=1}{\overset{m}{\max}}\underset{j=1}{\overset{n}{\max}}\left|{x^{\prime }}_{ij}-{y^{\prime }}_{j}\right|\right]}$$

where *ξ* denotes a discriminant coefficient within the range of 0 to 1, and generally the value of *ξ* is taken as 0.5 [60,62,63,64]. The grey relational grade (*γ_i_*) between *Y* and *X_i_* is formulated as

(5) $$\gamma_{i}=\frac{1}{n}\sum_{j=1}^{n}\gamma_{ij}$$

In this study, the sequence composed of the values of an index parameter (*β*) that denoting the *ε*_p_ versus *N* curve pattern was designated as the feature sequence *Y*_1_, where *β* values of 1.0, 2.0, and 3.0 were allotted to the stable, critical, and failure patterns, respectively, according to the recommendations of Javed et al. [65], Wu et al. [66], and Liu et al. [67]. In addition, the data sequences composed of moisture content *w*, confining pressure *σ*_3_, and dynamic deviator stress *σ*_d_ are termed as the observation sequences *X*_1_, *X*_2_, and *X*_3_, respectively. The computed values of the grey correlation coefficients *γ*_1*j*_, *γ*_2*j*_, and *γ*_3*j*_ are presented in Table 2, and the grey relational grades (*γ*_1_, *γ*_2_, and *γ*_3_) of *β* regarding *w*, *σ*_3_, and *σ*_d_ obtained by Equation (5) are 0.809, 0.745, and 0.720, respectively. Generally, the feature sequence is considered to be strongly correlated with the observation sequences if the calculated grey relational grade exceeds 0.6, as recommended by previous studies [62,63,64,68]. Therefore, it is considered that *β* (i.e., the curve pattern) is closely related to *w*, *σ*_3_, and *σ*_d_. In addition, slight differences appear among *γ*_1_, *γ*_2_, and *γ*_3_, implying that *w*, *σ*_3_, and *σ*_d_ have approximate importance degree on determining the *ε*_p_ versus *N* curve patterns of the UGM.

#### 4.1.2. Analysis of Specimen Pattern using the BP Neural Network

Most existing empirical models for the critical dynamic stress and cumulative plastic strain of subgrade soils are based on explicit analyses of limited test results. However, the *σ*_cri_ and *ε*_s_ of an UGM may have considerable non-linear correlations with influencing factors such as the *w*, *σ*_3_, and *σ*_d_, and the correlations are generally difficult to be identified by explicit analyses of the test results. In this study, the BP neural network was used to analyze the specimen pattern based on the MATLAB (version R2018b) platform. The MATLAB is a powerful and high-performance programming tool for technical computation, which integrates computation, visualization, and programming in a user-friendly environment [69]. The BP neural network generally consists of an input layer, several hidden layers, and an output layer, which exhibits strong non-linear mapping functions on multi parameters. Consequently, the BP neural network is appropriate for analyzing the *σ*_cri_ and *ε*_s_ of the UGM, and the developed empirical model is considered capable to effectively reflect the relationships of *σ*_cri_ and *ε*_s_ in regard to the *σ*_3_ and *σ*_d_.

**Figure 7:**
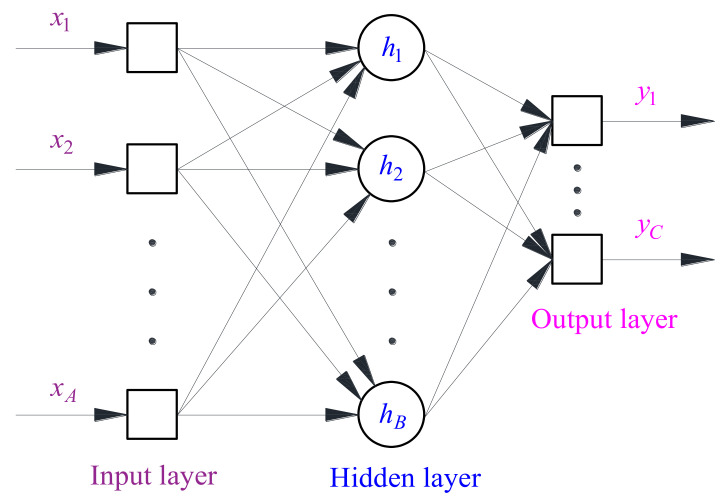
Topological graph of the BP neural network [16].

The BP neural network used in this study is a special machine learning method which focuses on intelligent predictions rather than improvements of computer algorithms, therefore the required amount of training data can be less than the traditional machine learning techniques, and a few studies have shown that training data with a size around 20 can yield acceptable training results [70,71,72,73,74]. The present study performed 28 cyclic triaxial tests on the UGM. Figure 7 presents a topological graph of a BP neural network that includes a hidden layer, in which the numbers of neurons in the input, hidden, and output layers are denoted by *A*, *B*, and *C*, respectively. The neurons in the input layer are specified as *w*, *σ*_3_, and *σ*_d_, while the neuron in the output layer is specified as the index parameter *β*, therefore *A* = 3 and *C* = 1 in the present study.

When the neurons in the input layer are assigned a data set regarding the *w*, *σ*_3_, or *σ*_d_, the neurons in the hidden and output layer(s) will generate values by following a transfer function, and the yielded values of the neuron in the output layer are the evaluated values of *β*. In the self-training of a BP neural network, the yielded values in the output layer (i.e., the evaluated values of *β*) generally exhibit deviations in comparison to the theoretical values or expectations. In this study, the expectations of *β* are 1.0, 2.0, and 3.0 regarding the stable, critical, and failure specimens, respectively. By inputting a series of data sets that correspond to different test conditions of *w*, *σ*_3_, or *σ*_d_, the computation of the BP neural network continues until the sum of deviations meets the required precision or the iteration number achieves the maximum. In this study, a sigmoid function was adopted for the transfer function, and the maximum of iterations is 2000. For more details regarding BP neural networks, one can refer to Rumelhart et al. [75].

**Figure 8:**
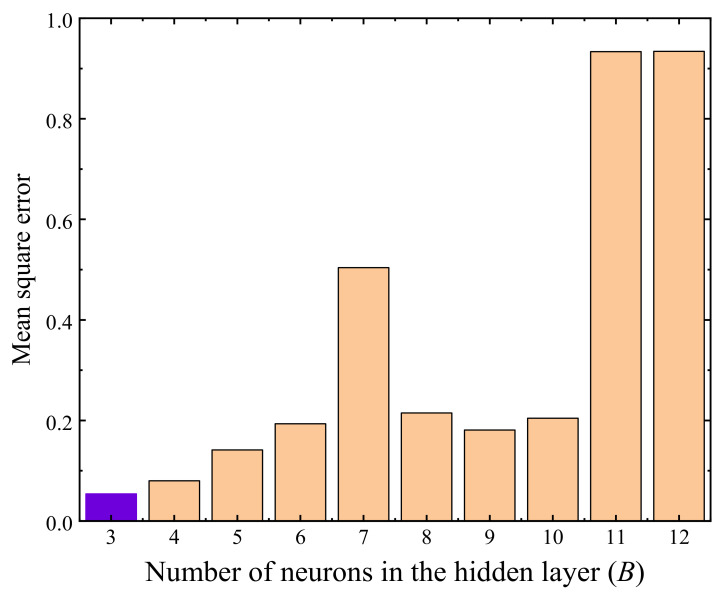
Mean square error with different *B* values (regarding the *σ*_cri_).

The number of neurons (i.e., the *B* value) in the hidden layer(s) is one of the key parameters that could considerably affect the trained neural network. Chen et al. [76] stated that the value range of *B* can be estimated as

(6) $$B=\sqrt{A+B}+S$$

where *S* is a constant that ranges between 1 and 10. The value range of *B* calculated by Equation (6) is 3–12. The mean square errors (MSE) of the trained BP neural network regarding the variation of parameter *B* are presented in Figure 8, where the results showed that the MSE attained the minimum value when *B* = 3, thus three neurons in the hidden layer were adopted in the following analyses.

**Figure 9:**
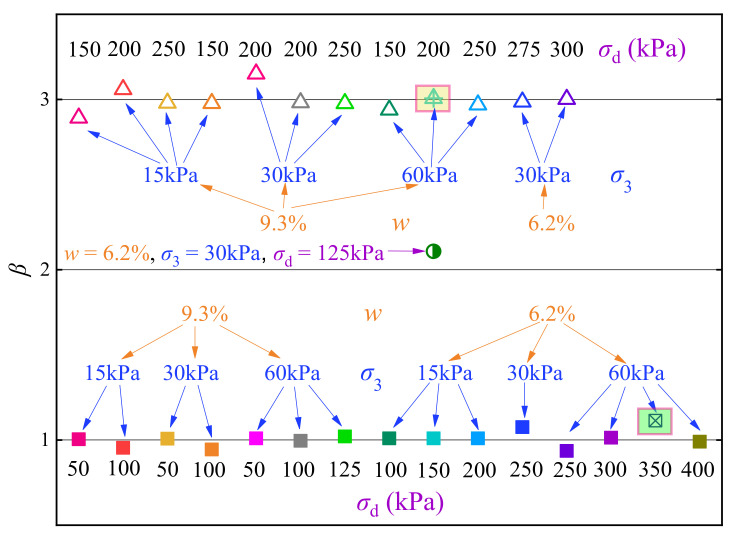
Output results of *β* using the trained BP neural network.

In training of the BP neural network, a stable specimen with test conditions of *w* = 6.2%, *σ*_3_ = 60 kPa, and *σ*_d_ = 350 kPa (corresponding to a *β* value of 1.0) and a failure specimen with conditions of *w* = 9.3%, *σ*_3_ = 60 kPa, and *σ*_d_ = 200 kPa (corresponding to a *β* value of 3.0) were excluded, which were used to verify the reliability of the trained BP neural network later on. Figure 9 presents the output of the trained optimal neural network using the remaining data (including the data of stable, failure, and critical specimens). The figure shows that the evaluated *β* values are close to theoretical values without data distortion or local minimum problems, and the predicted *β* values of the two specimens used to verify the trained neural network (data points in the rectangles) also agree well with the theoretical values, indicating that the trained optimal neural network is of acceptable reliability.

#### 4.1.3. Evaluation of Critical Dynamic Stress Using the Trained BP Neural Network

Evaluations of the *σ*_cri_ under different *σ*_3_ were implemented using the trained optimal BP neural network. Predetermined moisture contents and confining pressures were first input as the neuro values in the input layer. Then, the input neuron value with respect to *σ*_d_ is continuously changed until the *β* obtains 2.0. The final input *σ*_d_ which generates a *β* value of 2.0 is therefore the evaluated value of *σ*_cri_ with respect to the input *w* and *σ*_3_. The input *σ*_3_ is altered within 15–60 kPa by a 5 kPa interval, and the evaluated *σ*_cri_ values using the BP neural network are presented in Table 3.

**Figure 10:**
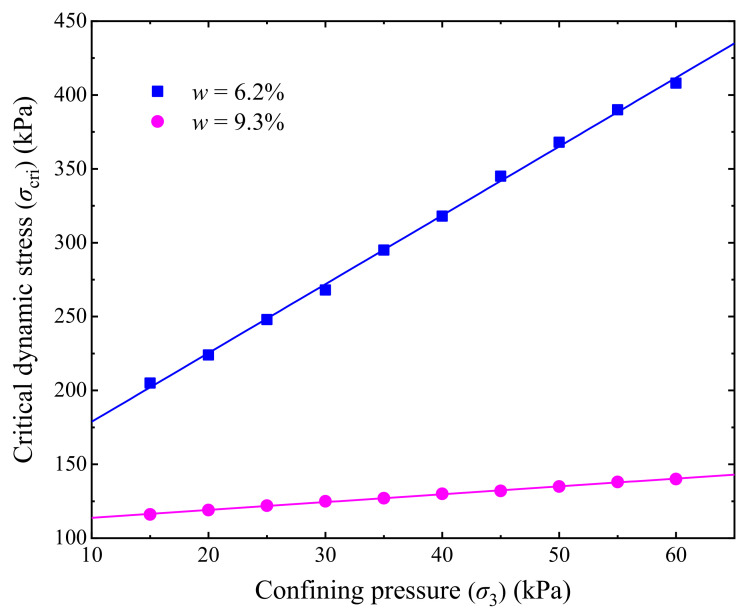
Relationship between critical dynamic stress and confining pressure.

Variations of the evaluated *σ*_cri_ of the UGM with the confining pressure are illustrated in Figure 10, with different lines corresponding to optimal and saturation moisture contents. It is found that the *σ*_cri_ approximately linearly increases as the confining pressure increases, and the correlation between *σ*_cri_ and *σ*_3_ can be formulated as Equation (7), where *m* and *n* are fitting parameters. Therefore, it is a feasible method to improve the dynamic stability of a railway subgrade by appropriate techniques (e.g., the prestressed embankment technique [6]) to increase the confining pressure of the subgrade soil. Figure 10 indicates that the *σ*_cri_ of the UGM is also influenced by the moisture content of the UGM. It is therefore considered that the model parameters *m* and *n* in Equation (7) could have correlations with the *w*, and Equation (7) can be reformulated as Equation (8), where *f*_1_(*w*) and *f*_2_(*w*) are functions of *w*. However, due to the limited moisture contents tested, the expressions of *f*_1_(*w*) and *f*_2_(*w*) are still not clear, and further investigations regarding this topic are desirable in future studies.

(7) $$\sigma_{\mathrm{cri}}=m\times \sigma_{3}+n$$

(8) $$\sigma_{\mathrm{cri}}=f_{1}\left(w\right)\times \sigma_{3}+f_{2}\left(w\right)$$

Dawson et al. [77] reported that the critical total vertical stress (*σ*_1d_, the sum of *σ*_cri_ and *σ*_3_) of unbound granular subgrade soils can be formulated as

(9) $$\sigma_{1d}=A{\left(\sigma_{1d}/\sigma_{3}\right)}^{B}$$

where *A* and *B* are model parameters.

Wang et al. [78] performed cyclic triaxial tests on an UGM, having a similar gradation, of which both the fine contents (less than 0.075 mm) of the materials tested by Wang et al. [78] and this study were around 10%. Wang et al. [78] reported that the values of model parameters *A* and *B* are 3838.8 and −1.583 for the UGM. Based on Equation (9), it is deduced that the correlation between the *σ*_cri_ and *σ*_3_ of the UGM is as

(10) $$\sigma_{\mathrm{cri}}=24.4123\times \sigma_{3}{}^{0.6129}-\sigma_{3}$$

**Figure 11:**
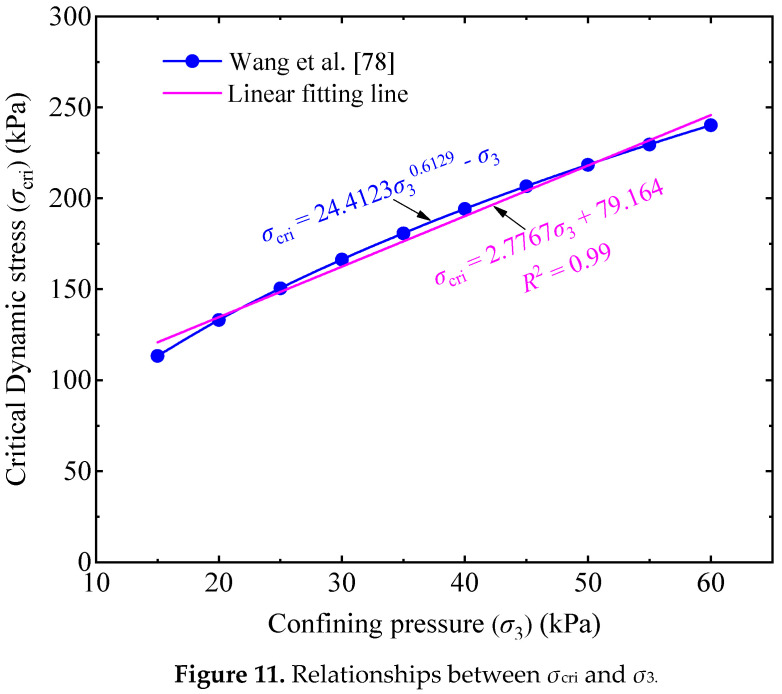
Relationships between *σ*_cri_ and *σ*_3_.

The functional image of Equation (10) is presented in Figure 11. It is observed that the relationship between *σ*_cri_ and *σ*_3_ shows some non-linearity; however, the non-linearity is not discernible when the UGM is in a low confining pressure service state, and can be approximately replaced by a linear relationship with a high determination coefficient (*R*^2^) of 0.99, as illustrated in Figure 11. Therefore, it is rational to simplify a linear relationship between *σ*_cri_ and *σ*_3_ of an UGM used as subgrade material.

### 4.2. Evaluation of Final Accumulative Plastic Strain

#### 4.2.1. Grey Relation of *ε*_s_ Regarding *w*, *σ*_3_, and *σ*_d_

A railway generally has strict requirements on surface deformation; therefore, the failure and critical patterns of the UGM are generally not expected in engineering practice. Consequently, this section primarily focuses on evaluating the final accumulative plastic strain *ε*_s_ of stable specimens (i.e., the class a curves in Figure 5 and Figure 6). The grey correlation theory was used to investigate the correlation degrees of *ε*_s_ in regard to *w*, *σ*_3_, and *σ*_d_ by taking the sequence composed of final accumulative plastic *ε*_s_ as the feature sequence *Y*_2_. The computed values of the grey correlation coefficients *γ*′_1*j*_, *γ*′_2*j*_, and *γ′*_3*j*_ are presented in Table 4, and the grey relational grades (*γ′*_1_, *γ*′_2_, and *γ*′_3_) of *ε*_s_ regarding *w*, *σ*_3_, and *σ*_d_ obtained by Equation (5) are 0.796, 0.733, and 0.724 (larger than 0.6), respectively; therefore, the *ε*_s_ is considered to be closely related to the *w*, *σ*_3_, and *σ*_d_. Furthermore, slight differences appear among *γ′*_1_, *γ′*_2_, and *γ*′_3_, implying that *w*, *σ*_3_, and *σ*_d_ have similar importance degree for the final accumulative plastic strain of the UGM. 

#### 4.2.2. Evaluation of Final Accumulative Plastic Strain Using the BP Neural Network

**Figure 12:**
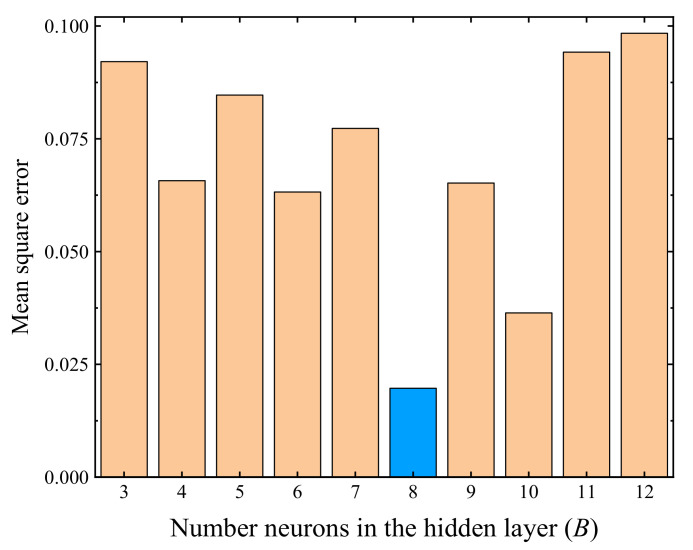
Mean square error with different *B* values (regarding the *ε*_s_).

**Figure 13:**
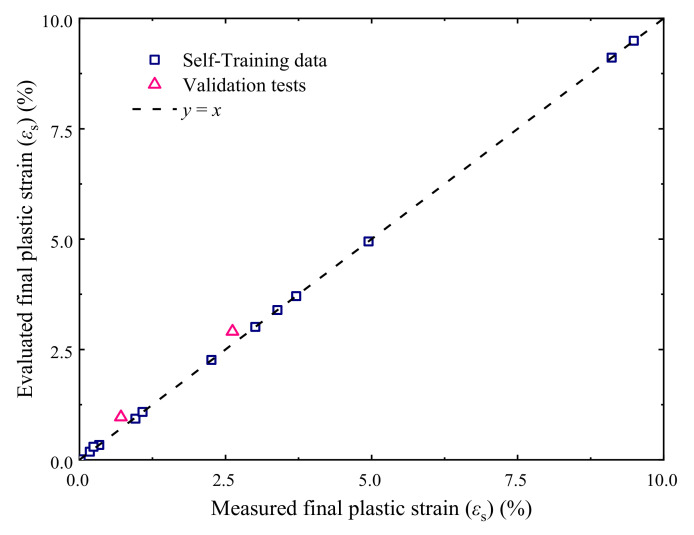
Output of *ε*_s_ using the optimal BP neural network.

The test data of stable specimens were used to perform the self-training of the BP neural network, excluding two specimens tested with conditions of *w* = 6.2%, *σ*_3_ = 60 kPa, and *σ*_d_ = 300 kPa (corresponding to a *ε*_s_ value of 2.62%) and *w* = 9.3%, *σ*_3_ = 30 kPa, and *σ*_d_ = 100 kPa (corresponding to a *ε*_s_ value of 0.71%), respectively, which were employed to verify the reliability of the trained BP neural network later on. The variation of the MSE of the trained BP neural network with the parameter *B* is presented in Figure 12, where the results show that the hidden layer of the optimal BP neural network contains eight neurons. Figure 13 presents the evaluated *ε*_s_ values using the trained optimal neural network, showing that the final accumulative plastic strains generated by the BP neural network agree well with the measured data, and the predicted *ε*_s_ of the two specimens used to verify the trained neural network also close to the measured values (see the triangular points in Figure 13), indicating that the trained optimal BP neural network is of acceptable reliability.

**Figure 14:**
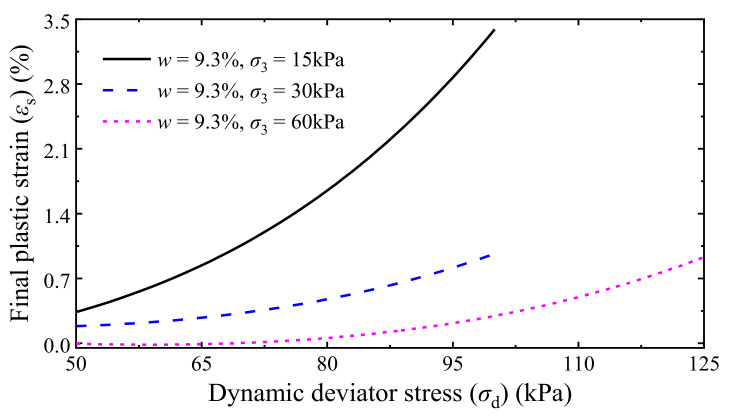
*ε*_s_–*σ*_d_ relationship of saturation specimens.

**Figure 15:**
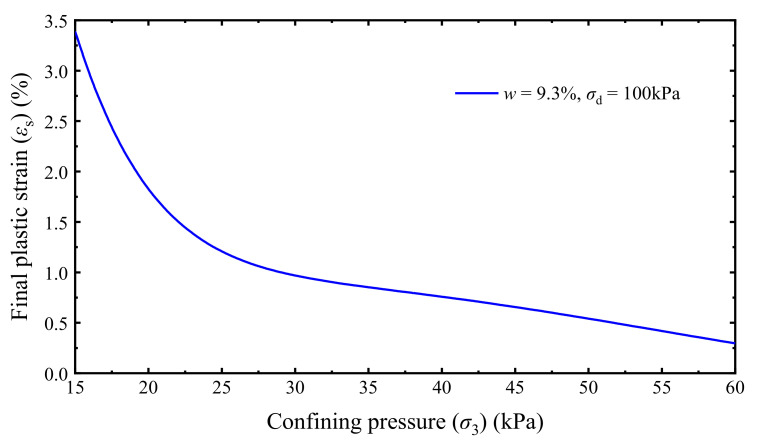
*ε*_s_-*σ*_3_ relationship of saturation specimens.

The *ε*_s_ of the UGM under different *σ*_3_ and *σ*_d_ can then be evaluated using the trained optimal BP neural network. The relationships of the predicted *ε*_s_ with respect to *σ*_d_ and *σ*_3_ are presented in Figure 14 and Figure 15, respectively. It can be found that *ε*_s_ and its variation rate non-linearly increase with increasing *σ*_d_ but non-linearly decrease as *σ*_3_ increases. Furthermore, the *ε*_s_ of saturation specimens with low confining pressures (e.g., 15 kPa in Figure 14) starts to increase in a large rate when the dynamic deviator stress exceeds 60 kPa. Freight trains operated in the heavy-haul railway system of China can generally impose a maximum dynamic stress greater than 60 kPa on the subgrade surface [17,57,79,80]. Therefore, keeping sufficient drainage capacity and preventing the subgrade approaching saturation are important in the routine maintenance of a railway. In addition, increasing the confining pressure can efficiently reduce the final accumulative plastic strain of the UGM, especially in low confining pressure conditions as illustrated in Figure 15.

## 5. Conclusions

The critical dynamic stress (*σ*_cri_) and accumulative plastic strain (*ε*_p_) are primary indicators regarding the dynamic stability of unbound granular materials (UGMs). In this study, the dynamic stability of a typical UGM employed in railway subgrade bed construction was investigated by performing a series of large-scale cyclic triaxial tests, while considering the variations of moisture contents (*w*), confining pressures (*σ*_3_), and dynamic deviator stresses (*σ*_d_). A data processing method in combination with a back-propagation (BP) neural network is developed to analyze the test results, and an effective method is presented to evaluate the *σ*_cri_ and the final *ε*_p_ (*ε*_s_) of the UGM. The main conclusions obtained are as follows:

- The variation curves of *ε*_p_ with cyclic number (*N*) can be categorized into stable, failure, and critical patterns, and the grey relational analyses shown that the curve patterns and final accumulative plastic strain *ε*_s_ of stable specimens are closely related to the *w*, *σ*_3_, and *σ*_d_. In addition, the analyzed grey relational grades were in a narrow range of 0.72 to 0.81, demonstrating that the *w*, *σ*_3_, and *σ*_d_ have similar importance degrees in determining the *ε*_p_ versus *N* curve patterns and the values of *ε*_p_ of the UGM.
- The developed evaluation method indicates that the *σ*_cri_ approximately linearly increases with increasing *σ*_3_, and a simple linear empirical equation is proposed for the *σ*_cri_. In addition, *ε*_s_ and its variation rate non-linearly increases with increasing *σ*_d_ but non-linearly decreases as *σ*_3_ increases; therefore, an increase in *σ*_3_ can effectively reduce the *ε*_s_, especially in low confining pressure conditions.
- The *ε*_s_ of saturation specimens with a low confining pressure of 15 kPa started to increase rapidly when the *σ*_d_ was greater than 60 kPa which is generally less than the dynamic deviator stress on subgrade surfaces imposed by freight trains operated in heavy-haul railway lines. Therefore, keeping sufficient drainage capacity, preventing the subgrade approaching saturation, and raising the confining pressure of the subgrade by appropriate reinforcement techniques are effective ways to enhance the railway dynamic stability.

## Figures and Tables

**Table 1:** Detailed program of cyclic triaxial tests.

Group	*w* (%)	*σ*_3_ (kPa)	*σ*_d_ (kPa)
1	9.3 (*S*_r_ = 100%)	15	50, 100, 125, 150, 200, 250
2	9.3 (*S*_r_ = 100%)	30	50, 100, 125, 150, 200, 250
3	9.3 (*S*_r_ = 100%)	60	50, 100, 125, 150, 200, 250
4	6.2 (*S*_r_ = 79.4%)	15	100, 150, 200
5	6.2 (*S*_r_ = 79.4%)	30	250, 275, 300
6	6.2 (*S*_r_ = 79.4%)	60	250, 300, 350, 400

**Table 2:** Grey relational coefficients of *β* with respect to *w*, *σ*_3_, and *σ*_d_.

*Β*	*σ*_3_ (kPa)	*σ*_d_ (kPa)	*w* (%)	*γ*_1j_	*γ*_2j_	*γ*_3j_
1	15	50	9.3	1.000	1.000	1.000
1	15	100	9.3	1.000	1.000	0.778
3	15	125	9.3	0.636	0.636	0.875
3	15	150	9.3	0.636	0.636	1.000
3	15	200	9.3	0.636	0.636	0.778
3	15	250	9.3	0.636	0.636	0.636
1	30	50	9.3	1.000	0.778	1.000
1	30	100	9.3	1.000	0.778	0.778
2	30	125	9.3	0.778	1.000	0.875
3	30	150	9.3	0.636	0.778	1.000
3	30	200	9.3	0.636	0.778	0.778
3	30	250	9.3	0.636	0.778	0.636
1	60	50	9.3	1.000	0.538	1.000
1	60	100	9.3	1.000	0.538	0.778
1	60	125	9.3	1.000	0.538	0.700
3	60	150	9.3	0.636	0.778	1.000
3	60	200	9.3	0.636	0.778	0.778
3	60	250	9.3	0.636	0.778	0.636
1	15	100	6.2	0.913	1.000	0.778
1	15	150	6.2	0.913	1.000	0.636
1	15	200	6.2	0.913	1.000	0.538
1	30	250	6.2	0.913	0.778	0.467
3	30	275	6.2	0.600	0.778	0.583
3	30	300	6.2	0.600	0.778	0.538
1	60	250	6.2	0.913	0.538	0.467
1	60	300	6.2	0.913	0.538	0.412
1	60	350	6.2	0.913	0.538	0.368
1	60	400	6.2	0.913	0.538	0.333
1	15	50	9.3	1.000	1.000	1.000
1	15	100	9.3	1.000	1.000	0.778

**Table 3:** Evaluated critical dynamic stress.

*w* (%)	*σ*_3_ (kPa)	*σ*_cri_ (kPa)	*w* (%)	*σ*_3_ (kPa)	*σ*_cri_ (kPa)	*w* (%)	*σ*_3_ (kPa)	*σ*_cri_ (kPa)	*w* (%)	*σ*_3_ (kPa)	*σ*_cri_ (kPa)
6.2	15	205	7.5	15	186	8.5	15	147	9.3	15	116
	20	224		20	203		20	155		20	119
	25	248		25	222		25	165		25	122
	30	268		30	242		30	177		30	125
	35	295		35	259		35	185		35	127
	40	318		40	277		40	194		40	130
	45	345		45	299		45	204		45	132
	50	368		50	319		50	214		50	135
	55	390		55	337		55	223		55	138
	60	408		60	357		60	235		60	140

**Table 4:** Grey correlation coefficients of *ε*_s_ with respect to *w*, *σ*_3_, and *σ*_d_.

*ε*_s_ (%)	*σ*_3_ (kPa)	*σ*_d_ (kPa)	*w* (%)	*γ′*_1*j*_	*γ′*_2*j*_	*γ′*_3*j*_
0.34	15	50	9.3	1.000	1.000	1.000
3.39	15	100	9.3	0.603	0.603	0.631
0.18	30	50	9.3	0.967	0.903	0.967
0.71	30	100	9.3	0.926	0.994	0.994
0.02	60	50	9.3	0.935	0.776	0.935
0.24	60	100	9.3	0.979	0.805	0.913
0.96	60	125	9.3	0.882	0.921	0.977
1.08	15	100	6.2	0.844	0.862	0.921
3.01	15	150	6.2	0.625	0.634	0.699
9.11	15	200	6.2	0.343	0.346	0.374
3.71	30	250	6.2	0.571	0.605	0.697
2.26	60	250	6.2	0.695	0.837	0.892
2.62	60	300	6.2	0.659	0.786	0.889
4.95	60	350	6.2	0.495	0.563	0.643
9.49	60	400	6.2	0.333	0.363	0.406

## Data Availability

The data used to support the findings of this study are available from the corresponding author upon reasonable request.
